# Application of 2D MoS_2_ Nanoflower for the Removal of Emerging Pollutants from Water

**DOI:** 10.1021/acsengineeringau.3c00032

**Published:** 2023-09-29

**Authors:** Bhavya Joshi, Ahmed M.E. Khalil, Shaowei Zhang, Fayyaz A. Memon, Zhuxian Yang

**Affiliations:** Faculty of Environment, Science and Economy, University of Exeter, Exeter EX4 4QF, U.K.

**Keywords:** Organic dyes, Ciprofloxacin, Emerging Contaminant, Transition metal dichalcogenides, Water treatment, Molybdenum disulfide, adsorbents

## Abstract

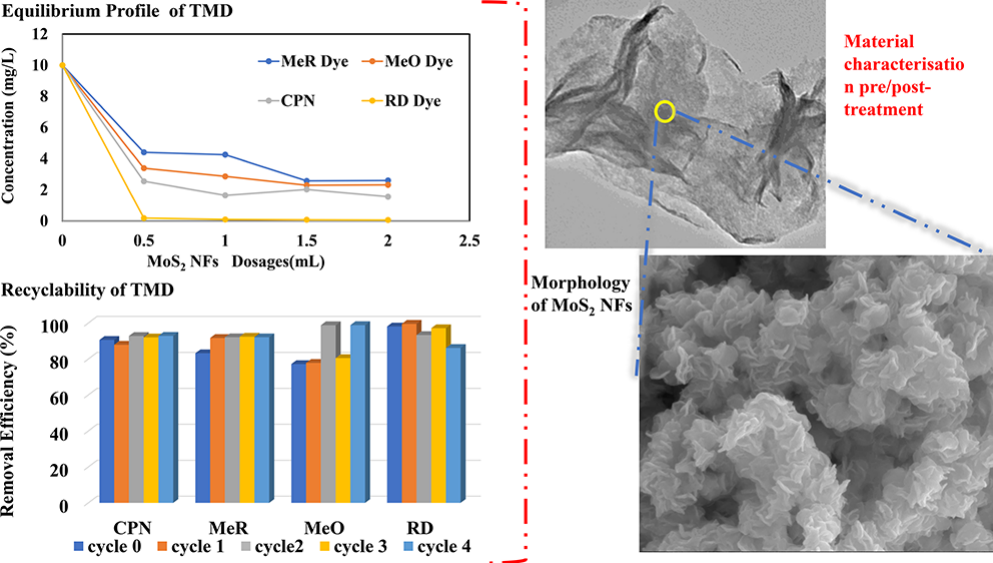

Two-dimensional (2D) nanomaterial-MoS_2_ (molybdenum disulfide) has gained interest among researchers, owing to its exceptional mechanical, biological, and physiochemical properties. This paper reports on the removal of organic dyes and an emerging contaminant, Ciprofloxacin, by a 2D MoS_2_ nanoflower as an adsorbent. The material was prepared by a green hydrothermal technique, and its high Brunauer-Emmett-Teller-specific area of 185.541m^2^/g contributed to the removal of 96% rhodamine-B dye and 85% Ciprofloxacin. Various characterizations, such as X-ray diffraction, scanning electron microscopy linked with energy-dispersive spectroscopy, and transmission electron microscopy, revealed the nanoflower structure with good crystallinity. The feasibility and efficacy of 2D MoS_2_ nanoflower as a promising adsorbent candidate for the removal of emerging pollutants was confirmed in-depth in batch investigations, such as the effects of adsorption time, MoS_2_ dosages, solution pH, and temperature. The adsorption mechanism was further investigated based on thermodynamic calculations, adsorption kinetics, and isotherm modeling. The results confirmed the exothermic nature of the enthalpy-driven adsorption as well as the fast kinetics and physisorption-controlled adsorption process. The recyclability potential of 2D MoS_2_ exceeds four regeneration recycles. MoS_2_ nanoflower has been shown to be an effective organic pollutant removal adsorbent in water treatment.

## 1 Introduction

Pharmaceuticals and industrial effluents are disposed of into freshwater at a very high rate, adversely affecting aquatic life and posing a threat to human health.^1−6^ A highly active and well-known class of pharmaceuticals—*fluoroquinolones* (FQs)—is commonly found in water.^7^ They include a wide range of antibiotics used in human and animal medicines.^8^ Ciprofloxacin (CPN) is one of the first generation FQs and was rated among the top ten pharmaceuticals found in wastewater,^4,9^ and it is commonly classified as one of the emerging contaminants. Therefore, its removal became a high priority in wastewater treatments. Several techniques were used to treat CPN,^10−16^ including photocatalytic degradation, ozonation, etc. Organic dyes, on the other hand, are a well-known class of pollutants for the environment and are deemed carcinogenic to human health.^17^ These dyes account for 15% of the world’s total dye production discharged as waste by fabrics and textile industries.^18^ Organic dyes such as Methyl orange (MeO), Methyl red (MeR), and rhodamine-B (RD) are commonly used in fabric dyeing processes. They are not immediately biodegradable and therefore remain in the water cycle/food chain for a long duration of time, risking life-threatening implications to the environment.^19^ RD dye, an azo dye, has the tendency to accumulate by microplastics and increase the potential risk to the environment.^20−22^ A variety of techniques was used to eliminate these dyes and CPN, such as adsorption,^23^ membrane filtration,^24^ flocculation,^25^ and reverse osmosis.^26^ Adsorption was considered the most effective removal method for oils, organic dyes, accumulation of ions, and emerging contaminants (ECs).^19,27^ Therefore, an effective and efficient adsorbent material is required for wastewater treatment applications to maintain ecological balance with the least amount of environmental impact.

Two-dimensional (2D) materials show great potential as adsorbent materials for water treatment applications. Among them, the carbon family has mostly been explored as an adsorbent material for removing several pollutants. Porous graphene (PG) was tested against six emerging contaminants: atenolol, carbamazepine, ciprofloxacin, diclofenac, gemfibrozil, and ibuprofen at their trace concentrations, and it showed an effective removal efficiency (>99%) at a low PG dosage (100 mg/L).^27^ It also demonstrated good recyclability and effective regeneration for up to 4 cycles.^27^ The removal efficiency of these pharmaceuticals was further increased using PG as a filter media in an adsorption column filter tertiary unit.^28^ On the other hand, composites such as GNP/BNA (graphene nanoplatelet/Boron Nitride) demonstrated a maximum adsorption capacity of 185 mg/g, in the removal of CPN.^29^ In addition, carbon nanotubes, activated carbon, clays,^30−32^ agricultural byproducts,^33,34^ and plant wastes were investigated as adsorbent materials for wastewater treatment. However, they exhibited slow adsorption kinetics, low yield, lack of adsorption selectivity, and poor recyclability and regeneration.^19^ Also, they came with the challenge of an expensive fabrication process and complexity in scaling up their production.^35^ Of these, the need for unconventional adsorbents is increasing.

Tenne et al. unfolded MoS_2_ (molybdenum disulfide) and WS_2_ (tungsten disulfide) nanotubes, 2D nanomaterials analogous to graphene,^36^ alternatively known as transition-metal dichalcogenide (TMD). They are 2D-semiconductor materials with exceptionally high electrical, optical, and mechanical properties. MoS_2_ is a 2D layered material with a hexagonal crystal structure that is composed of strong intralayer covalent bonds and weak interlayer van der Waals interactions.^37^ These materials exhibit various morphologies, such as nanotubes, nanosheets, nanoflakes, nanoflowers (NFs), and nanoparticles.^38,39^ Thermal reduction, high-temperature sulfurization, laser ablation, chemical vapor deposition (CVD), and sol–gel processes have all been investigated as ways to synthesize MoS_2_.^40−47^ Among the aforementioned methodologies, the solvothermal method has piqued the interest of many due to its broad potential in the synthesis of nanoparticles.^48^ MoS_2_ materials with different morphologies were the result of the above techniques and tested for various applications.^48^ They have recently been explored for water treatment applications, showing many promising results. MoS_2_ membranes, specifically their nanoporous, layer-stacked, and composite membranes, have been reviewed for industrial wastewater treatment, desalination, and antifouling properties.^49^ MoS_2_ nanosheets showed the potential to enhance membrane-based water treatment technologies.^49^ MoS_2_ nanoflowers demonstrated the fastest photocatalytic activity for the degradation of methylene blue and crystal violet dyes as well as excellent reproducibility.^50^ They also showed exceptional activity for the hydrogen evolution process even after 1000 cycles as well as greater stability and a favorable functional catalytic capability for a variety of applications.^51^ Apart from this, an ultrathin MoS_2_ nanoflower-based sensor demonstrated a 67% gas sensitivity with selectivity levels up to 10 ppm of NO_2_ at ambient temperature.^52^ Thus, MoS_2_ materials in their membranes, sheets, and particle morphologies have been investigated for use in water treatment applications. Other morphologies, however, remain open to further investigation and testing.

This work aimed to understand the efficacy of the emerging 2D MoS_2_ material with nanoflower morphology in water treatment applications. 2D MoS_2_ nanoflower was synthesized via a modified green hydrothermal-based technique^50^ and investigated as a promising adsorbent candidate for the removal of ciprofloxacin (CPN), methyl red (MeR), methyl orange (MeO), and rhodamine-B (RD) contaminants. This synthesis technique does not involve any carcinogenic acids or bases and is therefore considered greener than using highly volatile and toxic techniques such as CVD and laser ablation.^40−47^ The contaminants were tested under identical conditions, and isotherm models were analyzed to assist in understanding the adsorption process involved in their removal. Apart from these, the morphological and structural features of as-prepared 2D MoS_2_ nanoflower were investigated.

## 2 Materials and Methods

### 2.1 Preparation of Chemicals, Adsorbates, and Adsorbent

Thiourea (>99.0%) and ammonium molybdenum hydrate, MeO, MeR, and RD dyes, and analytical grade of pharmaceutical-CPN were acquired from Sigma-Aldrich Co. (Poole, Dorset, UK), and utilized as adsorbates without any additional purification.

2D MoS_2_ nanoflower was synthesized using a modified green hydrothermal technique, reported in ref (50), which is based on the nucleation, fusion, and Ostwald ripening process, using CH_4_N_2_S as a sulfur source.^50,53^ The modifications implemented are explained below.
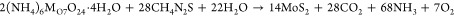
1

40 mL of deionized water was mixed with ammonium molybdenum hydrate {(NH_4_)_6_ MO_7_O_24_·4H_2_O} and thiourea (CH_4_N_2_S) and was mechanically stirred for 30 min to generate a clear solution. It was then placed into a 100 mL stainless steel autoclave and heated in an oven overnight at 180 °C (as schematically depicted in Supporting Information S1). After the reaction was finished, the autoclave was naturally cooled to room temperature (RT), and the resultant solution was sonicated for 30 m. The resulting black precipitate was centrifuged, extensively cleaned with ethanol five times, and then rinsed with water once more. The resultant black powder was further characterized after 15 h of drying at 90 °C before being used as an adsorbent in a water treatment test.

### 2.2 Phase Identification and Microstructural Characterization

A Bruker (D8 advanced) X-ray diffractometer was used to determine the phases of the materials using Cu K radiation at 40 kV and 40 mA. The diffraction patterns were collected across a range of 2–90° at a scan rate of 2° (2)/min.

The atomic bonds in the samples—such as the S–S and Mo–S bonds—were investigated using a Fourier transform infrared spectrometer (FTIR, Bruker Optics Tensor-27). The absorbed spectra were measured between 500 and 4000 cm^–1^ at a resolution of 4 cm^–1^ using 20 coadded images. 180 mg of potassium bromide (KBr) and 5 mg of each original sample were combined in a mortar and crushed under 5 MPa to make a pellet, which was then put in a sample container and examined in the FTIR’s optical chamber. A Jenway 6715 UV/vis spectrophotometer and a Renishaw Qontor Raman spectrometer (each with a 50× objective) were also used to record the samples’ UV–vis absorbance spectra and Raman spectra. In the latter case, the samples were made by spreading a small amount of powder onto a glass microscope slide and then flattening it. For each average, 10 spectra were collected using a 532 nm excitation laser with 5% power, a 20 s integration time, and background removal.

The microstructure and morphology of samples, including as-prepared and spent MoS_2_ particles, were examined using a scanning electron microscope (TESCAN VEGA3 SEM equipped with an X-MAXN EDS detector) in high vacuum at a voltage of 20 kV. The elemental compositions of phases in the samples were determined using energy-dispersive spectroscopy (EDS). The amount of residual adsorbent (TMD) after the first cycle of the water-treatment test was determined using morphological examination of wasted MoS_2_ particles. A JEOL-2100 tunnelling electron microscope (TEM) with a 200 kV accelerating voltage was used to perform high-resolution microstructural characterization of MoS_2_ samples. In this case, the sample was made by dispersing the prepared particles in ethanol, followed by drop-casting and drying the suspension with a micropipette on a copper grid covered in holey carbon. Using the N_2_ adsorption Brunauer–Emmett–Teller (BET) method, the specific surface area (SSA), porosity, pore volume, and pore size of the as-prepared MoS_2_ particles were determined. A Quantachrome Autosorb-iQ gas area characterization analyzer was used for the analysis. The sample was heated for 4 h at 200 °C to remove any contaminants that may have become trapped in the pores, and then it was chilled in an external bath at −195.8 °C. The nitrogen gas was then introduced into the sample chamber, which had been evacuated, where the pressure and overall volume were watched in order to determine the nitrogen adsorption–desorption isotherms.

### 2.3 Batch Tests

Batch tests were conducted for all four contaminants under different conditions. They were triplicated, and an average value was reported for each parameter. Further, the adsorption isotherm modeling (both linear and nonlinear regression) was done to assist in understanding the adsorption mechanisms.

The corresponding stock solutions were prepared using the pharmaceuticals, CPN, organic dye, MeO, MeR, and RD, along with distilled water (DW). They were stored in an airtight container and wrapped in aluminum foil to prevent photodegradation of the contaminants.

2D MoS_2_ suspensions were prepared by sonicating the powder with distilled water (DW) together for 30 min prior to batch tests.

#### 2.3.1 Effect of Contact Time

Kinetic studies were conducted for all pollutants using stock solutions with initial concentrations of 10.0 mg/L (MeR and MeO dyes) and 20.0 mg/L (CPN and RD dyes), for different time intervals at room temperature (22 ± 3 °C) without any pH adjustments of contaminant solutions (CPN, MeR, MeO, and RD dye). A 20 mL portion of each of these contaminant solutions was poured into a 50 mL airtight sealed bottle, followed by additions of a certain amount of adsorbent (2D MoS_2_) powder. For MeO and MeR, two different MoS_2_ dosages were added (1 and 0.5 mL injected from a 5-g adsorbent/L suspension), and for CPN and RD, a given amount of adsorbent was added (0.25 mL injected from a suspension of 5-g adsorbent/L). Each of the solutions was then magnetically stirred, and the samples were collected for the predetermined time period (*t* = 5, 10, 20, 40, 80, 120 min), after immediately being filtered using a 0.2 μm filter.

#### 2.3.2 Effect of Dosage of Adsorbent

Another batch test was performed to investigate the effects of varied adsorbent dosages (0.5, 1, 1.5, and 2 mL of adsorbent injected from a 5-g/L suspension) injected into a specific contaminant concentration (10.0 mg/L CPN and dyes). A 20 mL suspension of each contaminant with various adsorbent dosages was placed into a 50 mL centrifuge tube on a rotary shaker for 24 h, and then all samples for various MoS_2_ dosages were collected after being filtered using a 0.2 μm filter.

#### 2.3.3 Effects of pH and Temperature

At room temperature (22 ± 3 °C), batch adsorption tests were performed for all four contaminants under various pH and temperature conditions, in a 20 mL contaminant solution of 10.0 mg/L with added adsorbent (1.0 mL injected from a suspension of 5-g adsorbent/L). To demonstrate the pH effect, the initial pH of the contaminant solutions was altered to a specified value using 1 M NaOH or HCl solution. The pH of the solution remained unaltered even after treatment, and all suspensions with varying pH values (2–11) were stirred for 2 h at 200 rpm. The samples (in solution form) were collected by using a 0.2 m membrane filter. Similarly, to examine the effect of temperature, the pH of the solutions was kept at 7, and the test was performed at varying temperatures (15–80 °C) under the same conditions as before.

#### 2.3.4 Reusability Study

Four regeneration cycles were performed to assess the recyclability and reusability of as-prepared 2D MoS_2_ particles. A 50 mg adsorbent dosage was added to each of the four contaminants (each 200 mL, of 10.0 mg/L) and was kept for a 2 h contact period, at 200 rpm mechanical stirring speed at ambient temperature (22 ± 3 °C). The adsorbent particles were allowed to settle after sampling, and the supernatant was separated by using a peristaltic pump. DW was used to gently clean the used particles so that they could be used in the new adsorption cycle. This regenerated adsorbent substance was then put to use for four additional adsorption cycles while being subjected to the same adsorption conditions as fresh adsorbent. The outcomes supported both the exothermic characteristic of the enthalpy-driven adsorption process and the fast kinetics and the physisorption-controlled adsorption process.

## 3 Results and Discussion

### 3.1 Characterization of As-Prepared MoS_2_ Particles

**Figure 1:**
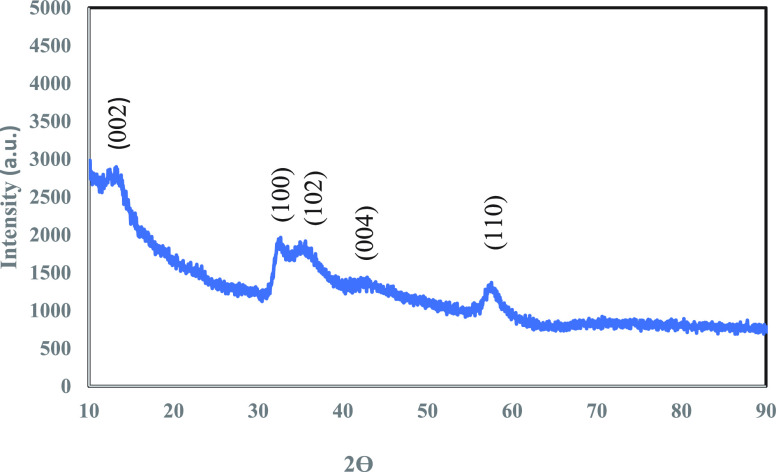
XRD pattern of 2D MoS_2_ particles synthesized by the green-hydrothermal technique.

Figure 1 depicts the XRD pattern of the as prepared 2D MoS_2_ particles. The peaks at 15.5, 32.3, 35.2, 43.2, and 57.21° correspond, respectively, to the (002), (100), (102), (104), and (110) 2D MoS_2_ hexagonal crystalline planes from JCPDS No. 37-492, respectively.^54−56^ The *d*-spacing for plane [002] was calculated to be 0.62 nm, which confirmed a crystalline structure of the 2D MoS_2_. Also, a low-intensity peak at plane [002] displayed a low stacking of the 2D MoS_2_ nanoflowers, which is further corroborated by SEM and TEM selected area electron diffraction (SAED) images. Apart from these, no other peaks appeared in the pattern, indicating the high phase purity of the as-prepared 2D MoS_2_.

**Figure 2:**
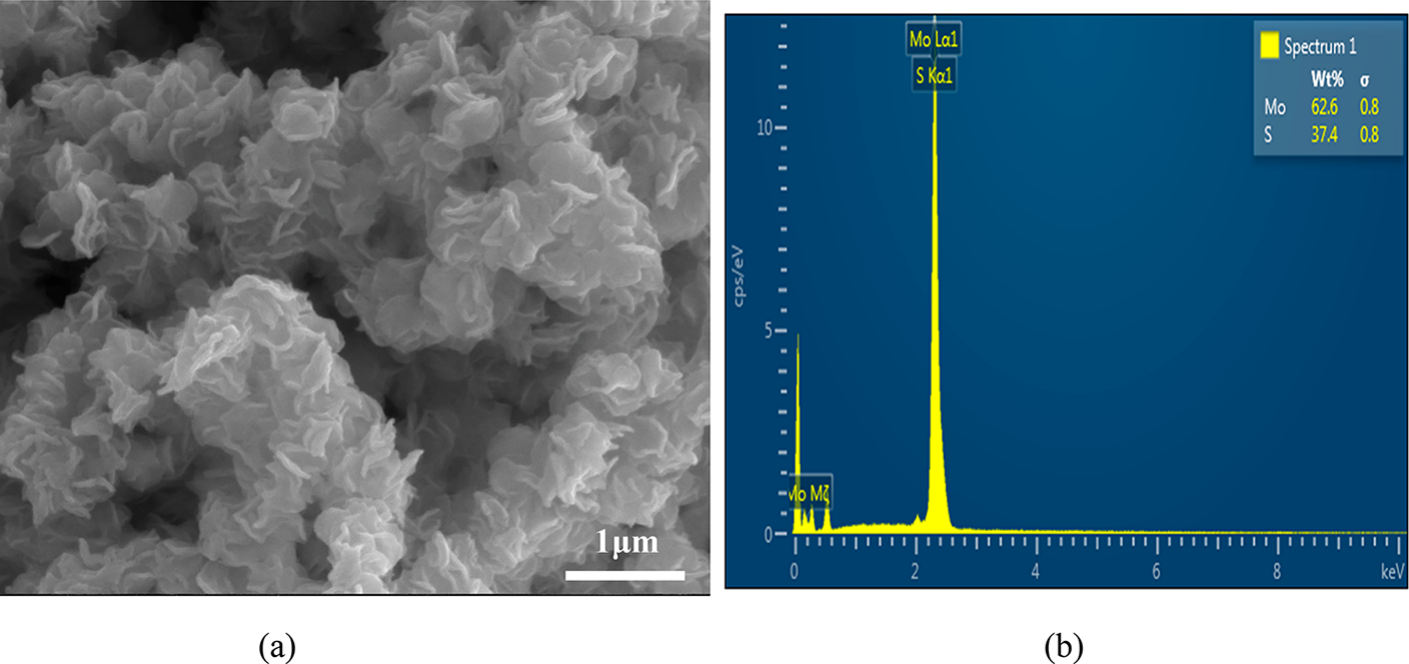
(a) SEM image of MoS_2_ NFs (synthesized by green-hydrothermal technique) at magnification 1 μm. (b) EDS spectra of 2D MoS_2_ NFs.

**Figure 3:**
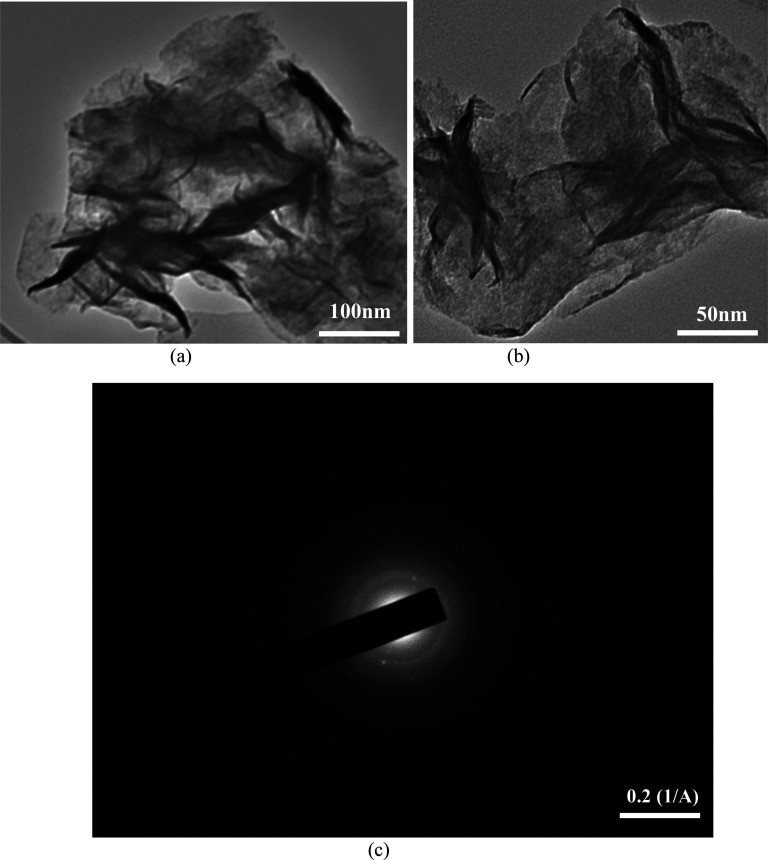
(a, b) TEM images at 50 and 100 nm scale and (c) SAED image of 2D MoS_2_ NFs (synthesized by green-hydrothermal technique).

The microstructure and morphology of as-received 2D MoS_2_ were characterized by SEM and TEM (with SAED pattern). SEM images of 2D MoS_2_ NFs, obtained at a lower scale (Figure 2 (a)), clearly depict the flower-like structure with minimum stacking in the edges of the as-synthesized material. This low stacking depicted in the 2D MoS_2_ SEM image validates the existence of the faint peak at the (002) plane found in XRD spectra. EDS spectra (Figure 2 (b)) were also acquired to understand the fraction of Mo and S atoms in the sample. TEM images of the sample prepared using thiourea as the sulfur source (Figure 3-a,b) were also obtained. The images showed nanopetals in a well-rounded layered structure and are connected to each other via narrow particle size with a regular spherical structure, which aids the SEM result.^50^ This shows that NFs have a uniform morphology as well as uniform particle size distribution. The SAED (Figure 3-c) pattern image of the 2D MoS_2_ NFs evidently showed several concentric diffraction circles with well-dotted lines corresponding to the 2D MoS_2_ planes and nanocrystalline nature, respectively.

As-prepared 2D MoS_2_ NFs UV–vis absorption spectra were also acquired and are displayed in Supporting Information-S2. The sample was investigated under the 250–700 nm spectra range with various dilution factors—100, 150, 200, 250 and 300, and the spectrum revealed a broad absorption peak located between 332 and 350 nm, clearly indicating the wide adsorption band for the bulk of 2D MoS_2_ NFs particles, which is in excellent agreement with the XRD result.^50^ Similarly, the FTIR spectrum of as-prepared 2D MoS_2_ (Supporting Information-S3) NFs displayed the bands at 602, 755, and 912 cm^–1^ arising from the Mo–S and S–S stretching.^48,57^ The hydroxyl group and the Mo–O vibration were assigned to the adsorption bands at 1100 and 1650 cm^–1^, respectively, demonstrating the presence of a hexagonal plane (002) in 2D MoS_2_ particles. This was consistent with the prior XRD and UV–vis results. Furthermore, the presence of the CH_2_ group (from unwashed residual thiourea and surface water stretching vibration) was responsible for the broad peak between 2982 and 3380 cm^–1^.^57,58^

The Raman spectrum of as-prepared 2D MoS_2_ (Supporting Information-S4) NFs showed vibrational modes at 378, 403, and 452 cm^–1^. The E_12g_ and A_1g_ peaks were formed due to the in-plane and out-of-plane vibrational modes of hexagonal 2D MoS_2_ between Mo–S and S–S, respectively.^52^ Raman spectra confirmed the presence of Mo–S and S–S bonds, which is consistent with the peaks identified in the XRD spectra (Figure 1) and confirms the synthesis of 2D MoS_2_ particles. All these characterizations confirmed the successful synthesis of 2D MoS_2_ NFs via a modified green hydrothermal technique.

**Figure 4:**
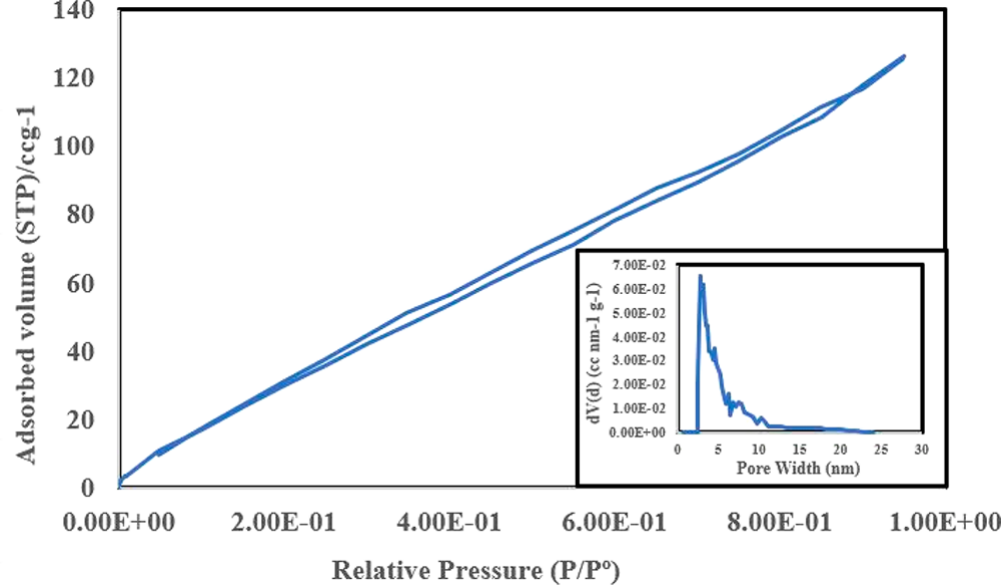
Measurement of the specific surface area. The pore size distribution of 2D MoS_2_ NFs was calculated using the Barrett–Joyner–Halenda method as an inset at 77 K, with pore size 3–5 nm.

The isotherms and Barret Joyner Halenda pore size distribution of 2D MoS_2_ NFs nanoparticles are depicted in Figure 4. The obtained isotherm of 2D MoS_2_ NFs depicts the formation of mono to multilayer nanopetals (as also shown in TEM and SEM images) and outlines the mesopores formation with pore size between 2 and 50 nm which corresponds to the IUPAC classified type (IV) adsorption isotherm.^59^ The presence of abundant active sites and high surface area contributes to the high adsorption capacity of the adsorbent for water treatment applications. Pristine 2D MoS_2_ exhibits an amorphous nature with BET-SSA ranging from 5.28 to 27.82 m^2^/g,^60^ depicting a lower removal potency as an adsorbent material. However, the BET-SSA of the investigated crystalline 2D MoS_2_ NFs was determined to be 185.541m^2^ /g, which was more than three times the 2D MoS_2_ NFs surface area (52.46 m^2^/g) obtained from the synthesis in ref (50). This proves that the modified green hydrothermal synthesis aided in increasing the BET SSA of the crystalline 2D MoS_2_ NFs. The faster increase in nitrogen isotherm at *P*/*P*_0_ depicted the formation of mesopores,^61,62^ with pore size between 3 and 5 nm, formed via reaction from thiourea, as a reductant material. Table S2 displays the remaining 2D MoS_2_ NFs sample parameters derived from the N_2_ adsorption–desorption isotherms.

The presence of stacking in the edges (as shown in Figure 2a SEM image) of the 2D MoS_2_ NFs acts as an intrinsic defect, creating more active sites, which aids the adsorption capacity of this crystalline adsorbent.^63^ Together with this higher BET-SSA, stacking edge fault, and layered nanopetal-like structure, the 2D MoS_2_ NFs provide a large and abundant active site for the adsorption of the tested organic contaminants, discussed in the next section.

### 3.2 Adsorption Performance

#### 3.2.1 Effect of Contact Time on Contaminants Adsorption

**Figure 5:**
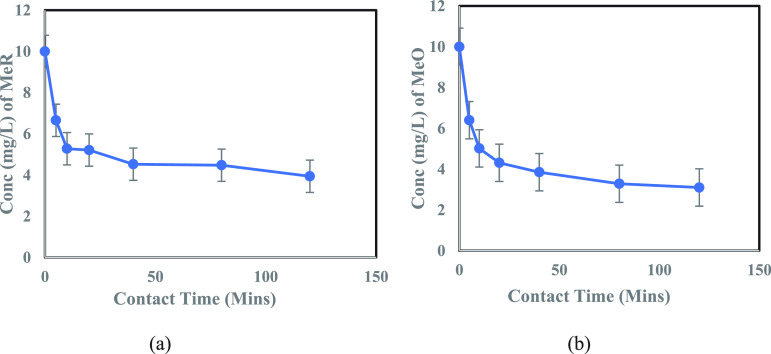
(a) Effect of contact time on adsorption of MeR (10.0 mg/L) onto 0.5 mL of TMD solution (5g/L). (b) Effect of contact time on adsorption of MeO (10.0 mg/L) onto 0.5 mL of TMD solution (5g/L).

**Figure 6:**
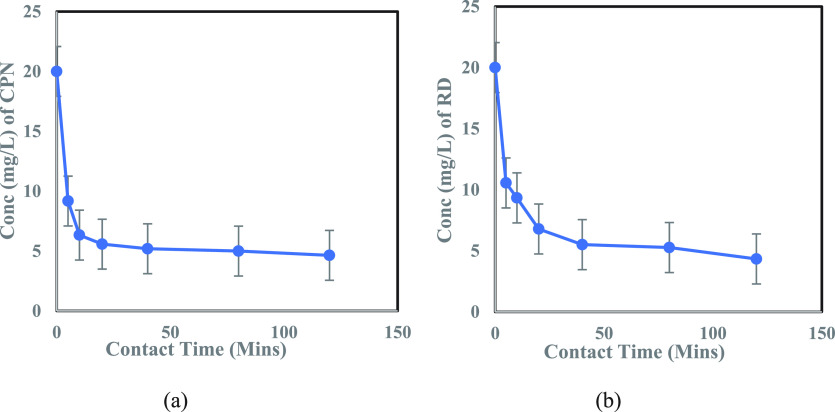
(a) Effect of contact time on adsorption of CPN (20.0 mg/L) onto 0.25 mL of TMD solution (5g/L). (b) Effect of contact time on adsorption of RD (20.0 mg/L) onto 0.25 mL of TMD solution (5g/L).

The adsorption rate is one of the most important parameters in batch tests. To investigate the influence of contact time on the adsorption of each of the four contaminants: CPN, MeO, MeR, and RD onto the adsorbent-MoS_2_ NFs, at room temperature, the contact time was varied from 5 to 120 m. The concentration vs contact time graphs for MeO and MeR dyes (10.0 mg/L concentration) with 0.5 mL of adsorbent dosage and for CPN and RD (20.0 mg/L concentration) with 0.25 mL of adsorbent dosage can be seen in Figures 5 and 6, respectively. 0.5 mL (injected from a suspension of 5-g adsorbent/L) of adsorbent dosage was used for all the cases, except for CPN and RD, as they showed extremely fast kinetics (Figure S6 (b)) for this dosage; therefore, for the sake of uniformity and better understanding of the kinetic modeling, 0.25 mL adsorbent dosage was considered for these two contaminants. For all four pollutants, fast sorption kinetics was observed in the initial stage of adsorption (40 min). This was due to the readily available high active sites (mesopores in nm size) on MoS_2_ petal-like surface (confirmed from TEM images), which served as an excellent trapping site for the contaminant particles, with diameter between 3.33 and 6 nm.^64,65^ Then, with the increase in time, the restricted availability of active sites decreased the adsorption prior to reaching saturation. The results indicated an excellent sorption performance of 2D MoS_2_ NFs (TMD), achieving 75–95% overall removal efficiency mainly due to the high specific area of 2D MoS_2_ NFs, as suggested by the BET analysis. Therefore, MoS_2_ NFs can be regarded as one of the 2D candidates for an adsorbent material, belonging to the noncarbon family.

Graphs, depicting the effect of contact time on the adsorption of MeR and MeO dyes onto the 2D MoS_2_ NFs, were also analyzed for 1 mL (injected from a suspension of 5-g adsorbent/L) adsorbent dosage (Figure S6(a)) and showed a similar kinetic trend as that for 0.25 mL of adsorbent dosage. To explain the reaction kinetics rate of the contaminants by MoS_2_ NFs, two models (pseudo-first-order and pseudo-second-order) (Supporting Information-S5)^66^ were examined to show the sorption kinetics.

The pseudo-second-order rate model fitted well for outlining the kinetics sorption of the contaminant onto MoS_2_ NFs, as demonstrated by the greatest regression correlation coefficient values (*R*^2^ = 0.99 for all four contaminants). This demonstrated a good linear relationship between *t*/*Q*_t_ and *t*,^66^ where “*Q*_t_” represents adsorption capacity at the moment “*t*”. Furthermore, the Qe (adsorption capacity at equilibrium) values estimated using the pseudo-second-order model were very close to the experimental Qe values.^67^ In this case, the adsorption rate is determined by the adsorption capacity rather than the adsorbate concentration. This demonstrates an excellent correlation with BET measurements, highlighting one of the adsorption rate and adsorption capacity correlations with the mesoporous sites present on the surface of MoS_2_ NFs. Table S3 lists all of the kinetic parameters with *R*^2^ values for both models. Adsorption kinetics can also be explained in terms of the pollutants’ and adsorbent’s hydrophobicity and hydrophilicity, as evidenced by the log value of the *n*-octanol/water partition coefficient (log *K*_ow_ values).^68^ It was proved that according to this indicator, CPN was a hydrophilic compound with more oxygen-containing groups.^27^ MoS_2_, generally in clean form, behaves hydrophilic in nature (at low water contact angles of ∼69 ± 3.8°),^68^ thus creating active sites for fast adsorption rate in the case of CPN (Figure S6(b)). However, a transition from hydrophilicity to hydrophobicity is observed in MoS_2_ (at high water contact angles of ∼89.0 ± 3.1°);^68^ i.e., in the presence of hydrocarbon contaminants, the hydrophilicity behavior of MoS_2_ renders into hydrophobicity.^69^ MoS_2_ in nanoflower morphology shows this transitional behavior,^70^ and thus we recorded the rapid kinetics for hydrophobic RD (Figure S6(b)). Similarly, the adsorption of MeO and MeR dyes onto MoS_2_ NFs (Figure 5) was owing to hydrophobic interactions, as MeO impurities are hydrophobic in nature,^70,71^ while MeR dye is somewhat hydrophilic/hydrophobic in nature.^72^ These hydrophobic interactions between hydrophobes and water enable hydrophobes (MoS_2_ NFs and hydrophobic) to be attracted to each other and orientated away from water.^27,73^ Therefore, the above discussion concluded that the adsorption rate for MoS_2_ NFs showed the dependency on its hydrophilic–hydrophobic nature and the active site present on the petal-like morphology of the crystalline 2D MoS_2_, obtained from TEM and BET characterization results.

#### 3.2.2 Effect of Adsorbent Dosages on a Certain Contaminant, in a Single Solution and Isotherm Modeling

**Figure 7:**
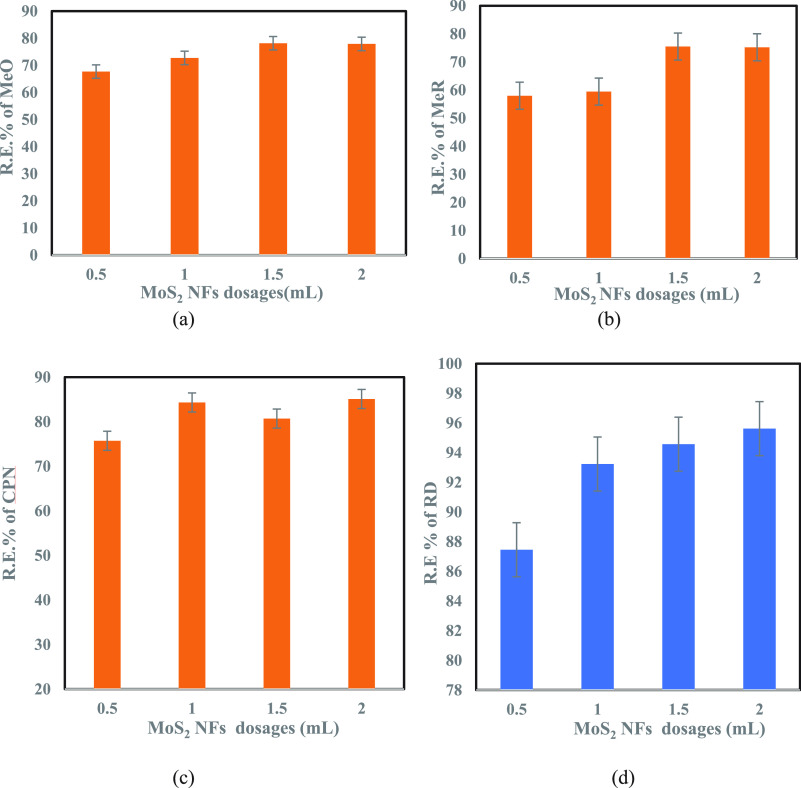
Equilibrium study for the four tested contaminants depicting their removal efficiency with varying MoS_2_ NFs dosages (mL): (a) MeO; (b) MeR; (c) CPN; (d) RD.

To understand the removal efficiency of MoS_2_ NFs adsorbent for these four contaminants, an adsorption batch test was conducted for different dosages of MoS_2_ NFs adsorbent (0.5, 1, 1.5, and 2 mL dosages of adsorbent injected from a 5-g/L suspension). Figure 7 reveals the removal efficiency of the adsorbent for these four contaminants with different adsorbent dosages. The adsorbent’s removal efficacy for the contaminants increased with its increasing dosages, with maximum removal efficiency observed at the highest dosage of MoS_2_ NFs adsorbent (2 mL dosage of adsorbent injected from a 5-g/L suspension). Of these three dyes—MeO, MeR, and RD—96% removal (calculated by Equation S1) was achieved for RD, and the rapid fading from color to colorless was observed for all dyes, whereas CPN revealed 85% removal by this adsorbent. A short summary of adsorbent removal efficiency versus adsorbent dosages for these four investigated contaminants are depicted in Table S1. Overall, a 75–96% removal efficiency was clearly achieved with this unconventional 2D adsorbent, MoS_2_ NFs. This excellent removal efficiency was likely due to the petal structure of MoS_2_ NFs (obtained from SEM and TEM results), which prevented coagulation and resulted in less flexibility of contaminant molecules on the surfaces.^74^ Therefore, this petal-like morphology favored the high removal of contaminants. The BET analysis, which revealed a high surface area and monolayer formation (from type IV isotherm), also contributed to this higher removal rate by MoS_2_ NFs. This demonstrates that MoS_2_ with this nanoflower morphology is an attractive candidate for wastewater treatment applications.

**Figure 8:**
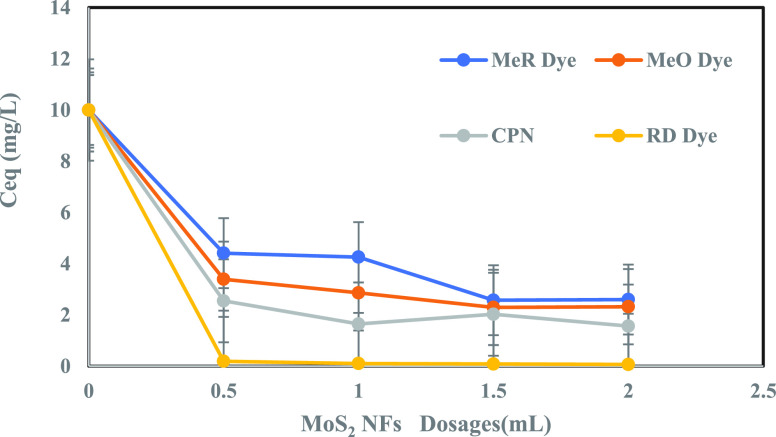
Equilibrium concentration (mg/L) for CPN, MeO, MeR, and RD dye Vs MoS_2_ NFs dosages (mL).

**Figure 9:**
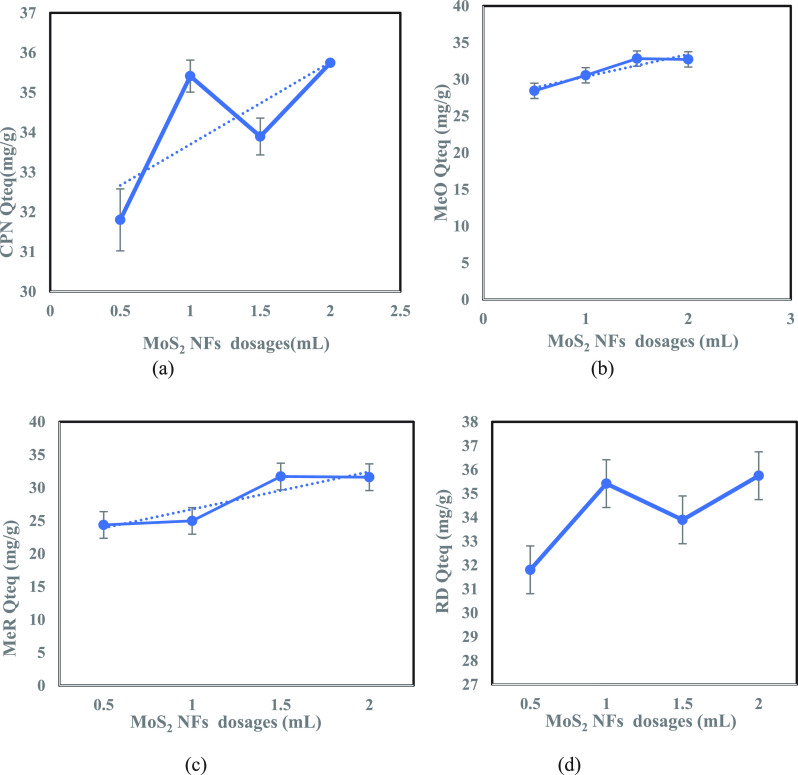
Adsorption curve for four tested contaminants at different MoS_2_ NFs dosages: (a) CPN; (b) MeO; (c) MeR; (d) RD dye.

After the equilibrium was reached, the concentration (*C*_eq_) of each of these four contaminants was obtained at varying dosages of MoS_2_ NFs (0.5, 1, 1.5, and 2 mL dosages of adsorbent injected from a 5-g/L suspension). Figure 8 demonstrates that upon reaching the equilibrium, the concentration for each of the four contaminants plummeted, as the adsorbent dosage was increased, indicating more adsorption at high adsorbent dosages. This was aided by MoS_2_ NFs adsorption capacity curves at different adsorbent dosages for the four contaminants: CPN, MeO, MeR, and RD (Figure 9). Contaminants CPN and RD revealed a similar zigzag adsorption curve with increasing adsorbent dosages. The highest adsorption capacity (37.74 mg/g for CPN and 35.74 mg/g for RD dye) was observed at a maximum adsorbent dosage, which was in line with the highest removal efficiency (RE) (Figure 7-c,d respectively). This higher adsorption capacity may be attributed toward the particle size of CPN and RD contaminants lying within the size MoS_2_ surface mesopores (3–5 nm, from BET analysis), contributing to more efficient trapping. MeO adsorption capacity curve (Figure 9-b) followed an increasing trend with different adsorbent dosages until 32.82 mg/g adsorption capacity was observed at 1.5 mL of adsorbent dosage. Furthermore, its adsorption capacity curve plateaued prior to a slight decrease, which was also reflected in its removal efficiency curve depicted in Figure 7-a. The adsorption curve for MeR (Figure 9-c) illustrated a gradual to sharp increase in adsorption capacity before reaching a saturation level with increasing adsorbent dosages. A very minimal variation was observed in adsorption capacity for the higher adsorbent dosages (31.70 and 31.59 mg/g for 1.5 and 2 mL of adsorbent dosages, respectively), which contributed to nearly identical removal efficiency for these two adsorbent dosages (Figure 7-b). When compared to RD dye (particle size 3.3 nm), MeO and MeR exhibit reduced adsorption because their particle sizes (6 nm) are somewhat larger than the mesopores of NFs, resulting in restricted trapping on these active sites.

For an understanding of adsorption behavior, adsorption isotherms are an important tool.^75^ In this work, we explored three models: Langmuir, Freundlich, and Temkin, in both linear and nonlinear forms.^66−77^ These models were able to express the adsorption process with the help of linear correlation coefficient *R*^2^ and other model parameters, which are listed in Table S4. In terms of *R*^2^ > 0.9 for linear modeling, Freundlich and Langmuir isotherms provided the greatest fit for all four pollutants. However, from the intrinsic parameters (linear regression-Table S4), the Langmuir model is more suitable to describe the adsorption mechanisms for MeO, MeR, and CPN contaminants due to their negative slope values obtained for Freundlich models.^78^ For RD dye, the negative intrinsic parameters (linear regression-Table S4) suggested that it follows neither of these models. The Temkin model for all four contaminants was ruled out due to its negative intrinsic parameter.^78,79^ The *R*^2^ value for all four contaminants is highlighted for the Freundlich and Langmuir isotherm models (Table S4).

**Figure 10:**
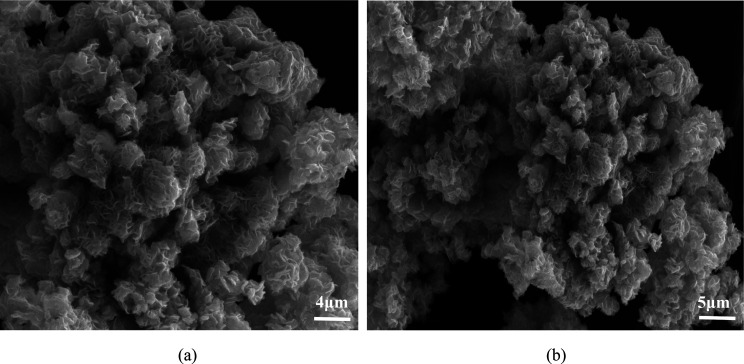
SEM image of the spent MoS_2_ NFs at different magnifications: (a) 4 μm; (b)5 μm.

Nonlinear modeling of these three adsorption isotherm models was also investigated,^77,78^ and results revealed that the Langmuir isotherm model fit the acquired experimental data the best. The lowest Akaike Information Criterion (AIC) values were obtained for this model (Table S5), for all four contaminants, confirming the equilibrium sorption process with a possibility of binary^80−82^ (both physio/chemisorption) sorption system. Moreover, for the Langmuir isotherm, the calculated/model adsorption capacity for all four contaminants was closer to the experimental adsorption capacity (listed in Tables S4 and S5). The Langmuir model fit was further confirmed by separation factors (*R*_L_) ranging between 0 and 1 for all four pollutants. The AIC values (Table S5), along with nonlinear model parameters (showed positive values), were calculated and highlighted for the best-suited model (Langmuir model). The Langmuir model predicts monolayer adsorption on the homogeneous surface of MoS_2_ NFs, corroborating the BET analysis’s finding of type IV isotherms forming mono to multilayers of MoS_2_ NFs. Additionally, the surface adsorption by the active sites (obtained from the BET study) of MoS_2_ NFs, which contributes to monolayer adsorption, is consistent with the Langmuir model’s predictions. Further investigation revealed that the SEM image (Figure 10, Supporting Information-S7) of the spent MoS_2_ NFs was in accordance with the Langmuir model and demonstrated the adsorption of these pollutants on monolayer surfaces without altering its flower-like morphology.

#### 3.2.3 Effect of pH on Contaminant Adsorption onto MoS_2_ NFs

**Figure 11:**
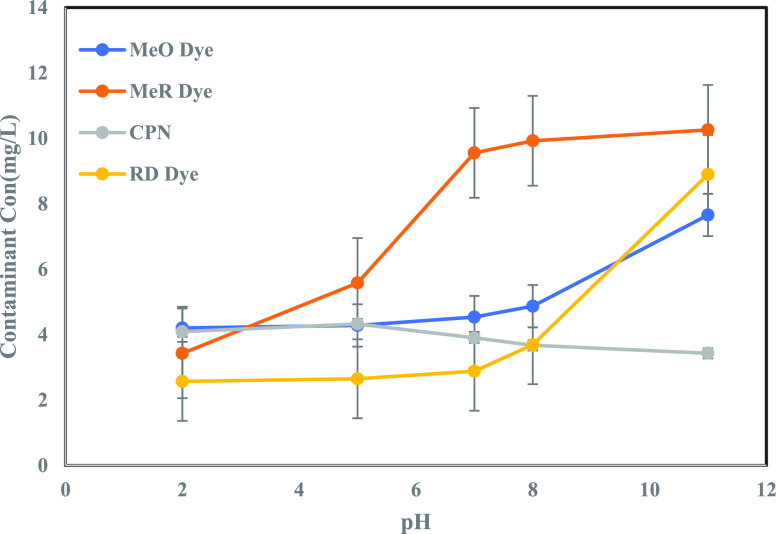
Effect of pH on each contaminant’s solution (conc. 10.0 mg/L) adsorption on 1 mL dosage of MoS_2_ NFs (5g/L).

The influence of pH on contaminant adsorption onto MoS_2_ NFs was studied over a broad range of the pH spectrum (2–11), and their concentration profiles are shown in Figure 11. All four contaminants showed individual and distinct profiles with pH.

MeO and RD dyes showed similar profiles. There was no significant change in the final concentration of pollutants (after 2 h) when the pH was very low (2 < pH < 4). However, when pH was above 8 (at the high alkaline condition), the final concentration of the contaminants showed a rapid increase favoring the desorption of these contaminants. This could be owing to the repulsion induced by electrostatic forces. At high pH, these petals of MoS_2_ NFs cause agglomeration resulting in aggregation of active sites, thus favoring desorption.^83,84^ MeR dye revealed a sharp increase in its final concentration in highly acidic conditions (pH < 5 and 5 < pH < 7) favoring desorption. Further desorption was seen when 7 < pH < 8, and no significant change was seen in its final concentration at pH > 8. The CPN concentration profile showed no notable change when pH < 5, followed by a rapid decrease in final concentration when 5 < pH < 8. Finally, the curve attained saturation when pH > 8. The highest removal of these four contaminants was seen for pH equal to 2. Except for greater performance and faster removal at pH > 7 for CPN, and desorption in the case of MeO and RD dyes, there was no significant difference found near neutral circumstances (for CPN, MeR, and RD dye). The desorption behavior observed was further used in the regeneration of spent MoS_2_ NFs and reused multiple times in water treatment. This investigation demonstrated that MoS_2_ NFs effectively remove these pollutants even under unfavorable pH ambient circumstances.

#### 3.2.4 Temperature Variation and Thermodynamic Modeling

**Figure 12:**
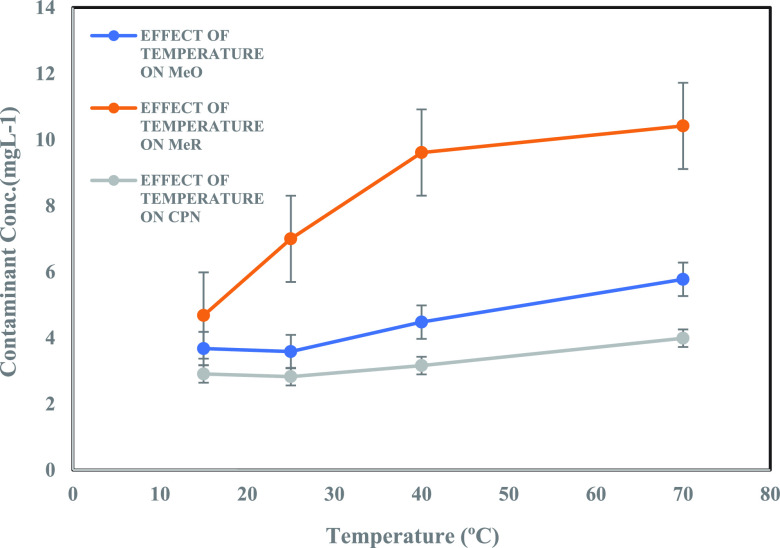
Effect of temperature on contaminant (10.0 mg/L) adsorption on 1 mL of adsorbent dosage (5g/L).

**Figure 13:**
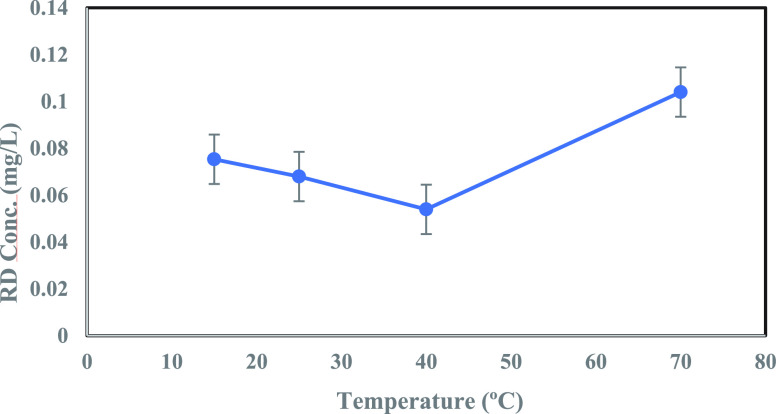
Effect of temperature on RD dye (10.0 mg/L) adsorption on 1 mL adsorbent dosage (5g/L).

The behavior of contaminants adsorbing onto MoS_2_ NFs adsorbent was investigated across a wide temperature range (15–70 °C). An optimal adsorbent dosage of 1 mL was taken into consideration for these contaminants, MeR, CPN, and RD, and the concentration profiles are depicted in Figures 12 and 13. Generally, with the increase in temperature, a notable increase in concentration was observed for MeR, MeO, and CPN contaminants, resulting in reduced absorption of these contaminants. On the contrary, increasing the temperature resulted in a considerable decrease in the concentration of RD dye. For all four contaminants, a rapid increase in concentration was seen at high temperatures (*T* > 40 °C). Thermodynamic parameters for these four contaminants were calculated according to the equation mentioned in refs (85−87) to understand the nature of the adsorption process. Curves were plotted between ln *K*_eq_ vs 1/*T* (Supporting Information-S8) with a correlation coefficient as shown in Tables S6–S9, along with the rest of the parameters of the model. Aside from the adsorption process’s nature, the model highlighted the process’s feasibility and spontaneity. The negative values of Δ*H* for all four contaminants confirm the exothermic nature of the adsorption, which clearly elucidates the decrease in adsorption behavior at high temperatures. This shows that the adsorption of MoS_2_ NFs for these contaminants has some selectivity, which could be further explored for water remediation applications. The Gibbs free energy change Δ*G* determines the spontaneity (sign-dependent) and nature (magnitude-dependent) of the removal process. Δ*G* is negative for three contaminants—CPN, MeO, and RD—indicating a spontaneous behavior, and it showed a positive value at higher temperatures for MeR dye, indicating nonspontaneous behavior at higher temperatures. It is believed that the adsorption process is driven by physisorption when 2 kJ mol^–1^ < Δ*G*° < 20 kJ mol^–1^,^85^ which is obtained for all four contaminants. Therefore, we can say that all four contaminants showed possible involvement in the physisorption removal process. The positive sign of Δ*S* in the case of RD represented the high randomness. On the contrary, a decrease in entropy (negative Δ*S*) was observed for the rest of the three contaminants. It was stated that when the reaction is exothermic but experiences a decrease in entropy, then the overall “enthalpy” favors the reaction.^85^ So, we can easily derive from this that all four contaminants involved physisorption in the removal process.

#### 3.2.5 Recyclability of MoS_2_ NFs

**Figure 14:**
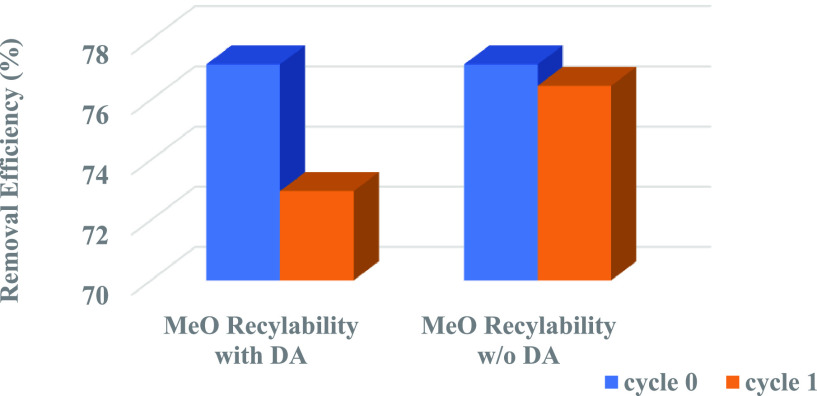
Recyclability of MoS_2_ NFs for the adsorption of MeO dye, with and without the desorption agent NaOH (DA).

To explore the reusability of MoS_2_ NFs adsorbents, a recyclability test was carried out. The conditions were similar to the previous investigation except for the dosage of MoS_2_ NFs (50 mg) and volume of contaminants (200 mL), which had been stated above in the Materials and Methods section. The MoS_2_ NFs were recycled four times after washing repeatedly with deionized water. The regeneration via heating at 90 °C with basic 1 M NaOH (as a desorption agent-DA) was also tried for MeO dye (Figure 14), but a decrease in the MeO removal from 76% to 72% was observed when a DA (at temperature 22 ± 3 °C) was applied. Also, the reactions—after the first cycle with DA (at temperature 22 ± 3 °C) and after first wash with DA (at temperature 90 °C)—were terminated, as the solution turned orange-yellow, leaving no MoS_2_ NFs to be carried on for the next regeneration cycle (Supporting Information S7). This was probably due to the formation of a crystalline salt (sodium molybdate), an inorganic compound of sodium sulfate or inorganic sodium salt, when MoS_2_ NFs completely reacted with NaOH (eq 2). Hence, the desorption agent NaOH was not used for the recyclability experiment.
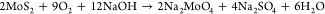
2

**Figure 15:**
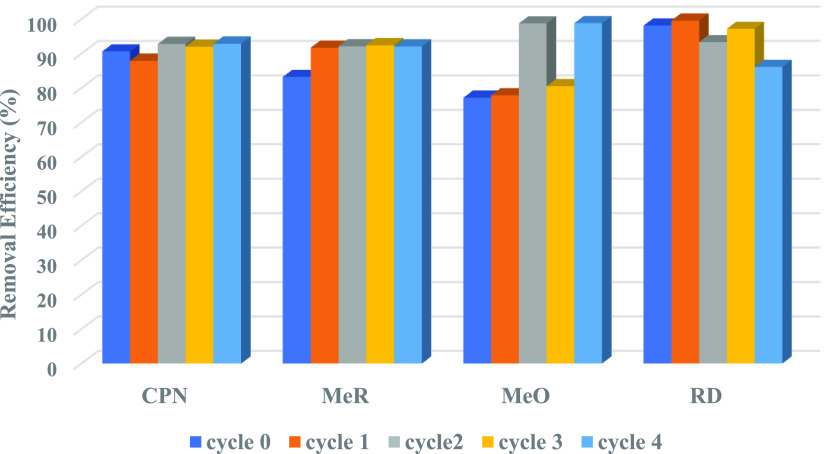
Recyclability of MoS_2_ NFs for the adsorption of contaminants.

The recyclability of MoS_2_ NFs on the contaminant’s adsorption process is depicted in Figure 15. Without any major loss, the MoS_2_ NFs showed a comparable removal capability with recyclability for up to 4 cycles. This was probably due to the flower-like morphology of MoS_2_, which exposed sufficient nanopetal intercalations on the surface and greatly enhanced the active sites on the edges,^74^ as well as the involvement of physisorption in the removal process, which made retrieval of MoS_2_ NFs much easier than involvement of chemisorption. This flower-like morphology of MoS_2_ was considered best for the degradation of dyes^74^ and was in line with the SEM-EDS analysis for the spent MoS_2_ NF (Supporting Information S7). A minor decrease in the removal efficiency of MoS_2_ NFs for these pollutants was most likely caused by an inadequate regeneration process (without the use of any DA); therefore, there is a need for a good adsorption agent for MoS_2_ NFs in water treatment applications.

Table 1 summarizes the various adsorbents in ascending order of their BET-SSA, for water treatment applications. The 2D MoS_2_ NFs, synthesized in this research, had the third highest BET-SSA, when compared with adsorbents belonging to the conventional carbon family, and exhibited a similar or higher adsorption capacity for CPN and dyes. Mesoporous SBA-15, having amorphous morphology, showed higher BET-SSA than the crystalline 2D MoS_2_ NFs; however, it was unable to remove the organic dyes and CPN and showed poor adsorption capacity for the rest of the emerging contaminants. Red-amber-green (RAG), of the synthesis process, is also depicted in the table, demonstrating the environmentally friendly nature of the synthesis process. Of all the synthesis processes describing their RAG to be Green (G), 2D MoS_2_ NF is the only adsorbent with high BET-SSA, belonging to the noncarbon family of the adsorbents. Therefore, these adsorbents can be an easy and facile alternative to many adsorbents belonging to the carbon family, with comparable removal efficacy toward emerging pollutants removal.

**Table 1:** Comparison Table of the Adsorbents Depicted in Ascending Order of Their BET SSA, for Water Treatment Application

					**RAG of Synthesis Process**
					*R-red*
					*A-Amber*
**ADSORBENT MATERIAL**	**BET SSA (m**^**2**^**/g)**	**Contaminants Tested for Adsorption/Absorbate**	**ADSORPTION CAPACITY**(mg/g)	**References**	*G-Green*
GRAPHITE	4.5	Carbamazepine-CBZ		(88)	G
		Methyl Orange- MeO	CBZ- 3.65 ± 0.05	(89)	
			MeO- 13.6	(90)	
Traditional MXenes	8.9	Rhodamine-b	RD- 22	(90)	R
Commercial Graphene	15	Carbamazepine-CBZ		(91)	A
		Methyl Orange- MeO			
		Ciprofloxacin-CPN	CPN- 323	(92)	
		Atenolol-ATL	ATL- < 6	(93)	
		Ibuprofen-IBP	IBP- 6.0	(93)	
		-BR	CBZ- 22.8 ± 0.5	(94)	
			BR 46- 30.52	(95)	
			MeO- 89.3	(96)	
Chemically reduced Graphene Oxide (rGO)	53			(97)	R
		Carbamazepine-CBZ			
		Methyl Orange- MeO	CPN- 18.2	(98)	
		Diclofenac sodium -DCFS,	DCFS- 59.67	(99)	
		Malachite Green -MG	CBZ- 55.13	(100)	
			MG- 279.85	(101)	
			MeO- 244	(102)	
Bare NZVIs	62			(103)	A,R
		Methyl Orange- MeO	MeO- 1.89 [Est.]	(104)	
Commercial Porous Graphene (PG)	82.76				A
		Carbamazepine-CBZ			
		Methyl Orange- MeO	CPN- 11.34		
		Methyl Red- MeR	CBZ- 7.92		
		Ciprofloxacin-CPN	ATL- 0.84		
		Atenolol-ATL	IBP- 3.976	(105)	
		Ibuprofen-IBP	MeO- 7.06		
		Rhodamine b-RD	MeR- 13.48		
			RD- 4.872		
MWCNT	160	Carbamazepine-CBZ		(106)	A,R
		Methyl Orange- MeO			
		Ibuprofen-IBP	CPN- 1.745	(107)	
		Ciprofloxacin-CPN	CBZ- 108	(108)	
			MeO- 27.6	(109)	
			IBP-186.5	(110)	
TMD-2D MoS_2_ NFs	185.54	Ciprofloxacin-CPN		This paper	G
		Methyl Orange- MeO	CPN- 35.74		
		Methyl Red- MeR	MeO- 32.71		
		Rhodamine b-RD	MeR- 31.70		
			RD- 41.73		
GREENER PG	289.14	Ciprofloxacin-CPN	CPN- 28.596		G
		Methyl Orange- MeO	CBZ- 25.74		
		Methyl Red- MeR	ATL- 19.29		
		Rhodamine b-RD	IBP- 37.65		
		Atenolol-ATL	MeO- 37.16	(105)	
		Ibuprofen-IBP	MeR- 84.75		
		Carbamazepine-CBZ	RD- 38.52		
Mesoporous SBA-15	737	Ibuprofen-IBP	IBP- 0.34		A
		Carbamazepine-CBZ	DCF- 0.07	(111)	
		Diclofenac-DCF	CBZ- 0.16		

Thus, this research investigated and summarized a potential nonconventional application with an adsorbent candidate having excellent recyclability for the removal of emerging contaminants in water treatment applications.

## 4 Conclusion and Outlook

This study aimed to explore an unconventional 2D MoS_2_ in its flower-like form (MoS_2_ NFs) to be a potential adsorbent candidate for wastewater treatment applications. MoS_2_ NFs were synthesized via the modified green hydrothermal method, and the removal efficiency of MoS_2_ NFs was tested against four contaminants: CPN, MeO, MeR, and RD dye. The structure and morphology of the material were tested and confirmed by various characterization tools such as XRD, SEM-EDS, FTIR, UV–vis, TEM, and BET analysis. Adsorption performances were elucidated, and concentration profiles were obtained for different batch conditions, (i) contact time, (ii) adsorbent dosages, (iii) pH and temperature effects, and finally reusability of MoS_2_ NFs was also examined. Adsorption modeling was done to describe the removal process of these contaminants. To eliminate errors, linear and nonlinear regressions were used. The study revealed: (i) rapid adsorption kinetics and a model that adhered to a pseudo-second-order; (ii) adsorption of the contaminants increased with the increase in the adsorbent dosage; (iii) the Langmuir isotherm model (according to lowest AIC values) revealed the favorability of equilibrium sorption process with a possibility of binary sorption system; (iii) thermodynamic modeling later confirmed the exothermic nature of adsorption process, governed by physisorption only; (iv) good reusability of MoS_2_ NFs for up to 4 cycles. Based on these experimental results and modeling isotherms, MoS_2_ NFs can be considered nonconventional adsorbent candidates for water treatment applications.

The majority of the research for 2D MoS_2_ focuses on the photocatalytic degradation of the pollutants. MoS_2_ NF/carbon fiber was investigated for piezocatalytic filters to recycle the decomposed wastewater and showed complete degradation up to 3 cycles.^112,113^ Until now, no study has examined MoS_2_ NF’s recyclability in water treatment applications. However, further optimization methods, such as the necessity for a good desorption agent for MoS_2_ NFs and the search for optimal conditions for MoS_2_ NF regeneration for a more cost-effective recycling process, could be adopted in future studies. These could be directions worthy for future works.

## Supporting Information Available

UV–vis result, FTIR result, Raman result, Kinetic modeling, Thermodynamic modeling, Spent SEM result for RD dye (PDF)
