# Supplementary material for: Application of 2D MoS2 Nanoflower for the Removal of Emerging Pollutants from Water
Source: ACS Eng Au. 2023 Sep 29;3(6):461–76. doi: 10.1021/acsengineeringau.3c00032 (PMC10739627; doi:10.1021/acsengineeringau.3c00032)
Supplement: Supplementary file 1 — eg3c00032_si_001.pdf [file eg3c00032_si_001.pdf]

APPLICATION OF 2D MoS_2 NANOFLOWER FOR THE REMOVAL OF EMERGING POLLUTANTS FROM WATER

Bhavya Joshi[a*], Ahmed M.E. Khalil[a], Shaowei Zhang[a*], Fayyaz A. Memon[a], Zhuxian Yang[a]

[a] Faculty of Environment, Science and Economy, University of Exeter, UK EX4 4QF

*Corresponding authors' e-mail address: bj300@exeter.ac.uk, s.zhang@exeter.ac.uk

S1. Synthesis of 2D MOS_2 nanoflower

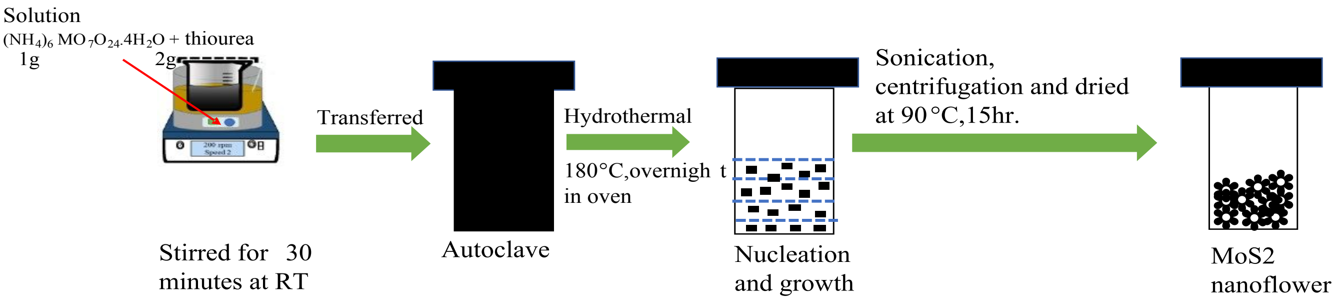

Figure S1: Schematic illustration of TMDs (MoS_2 nanoflower in powder form) obtained from green- hydrothermal method.

S2. UV-vis result of MoS_2 nanoflower

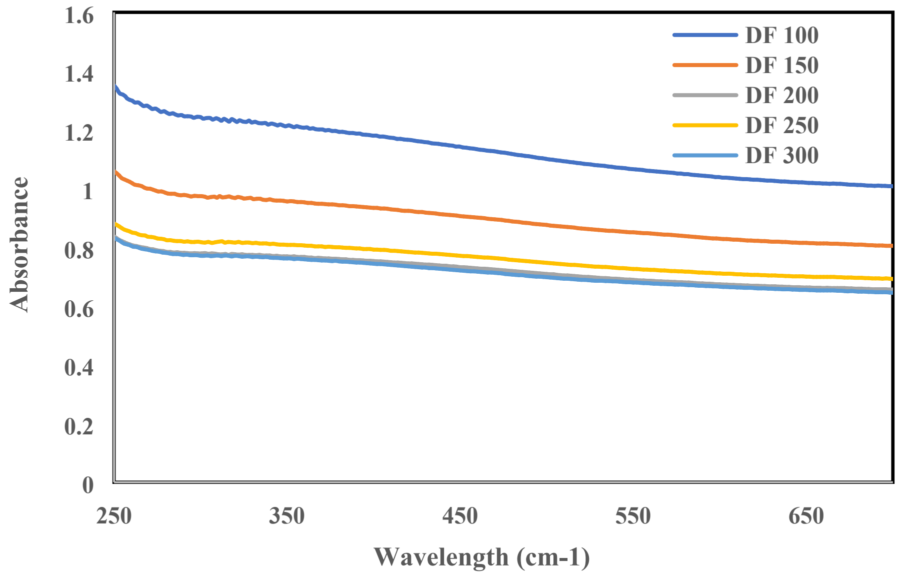

Figure S2: UV-vis spectra of MoS_2 nanoflower dispersion in distilled water at various dilution factor.

S3. FTIR result of MoS_2 NFs.

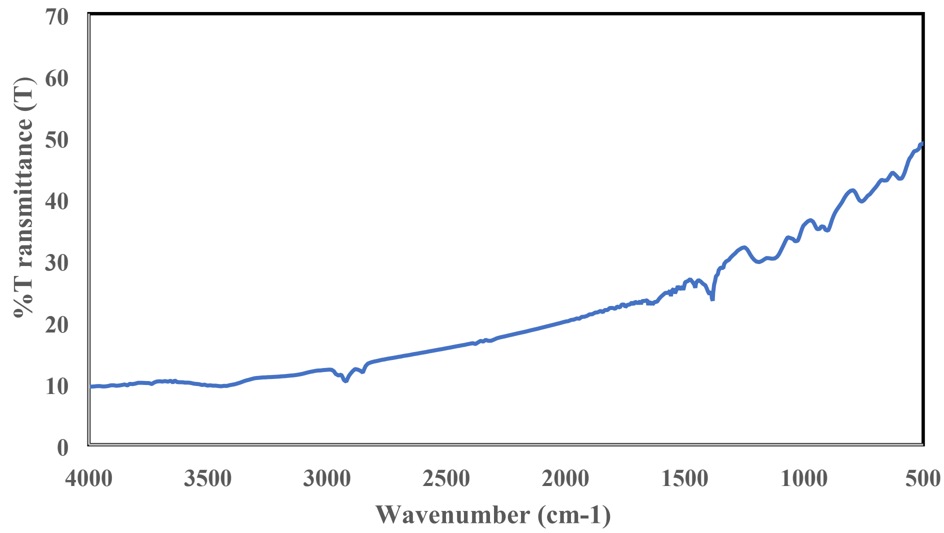

Figure S3: FTIR spectra of MoS_2 NFs.

S4. Raman Spectrum- MoS_2 NFs

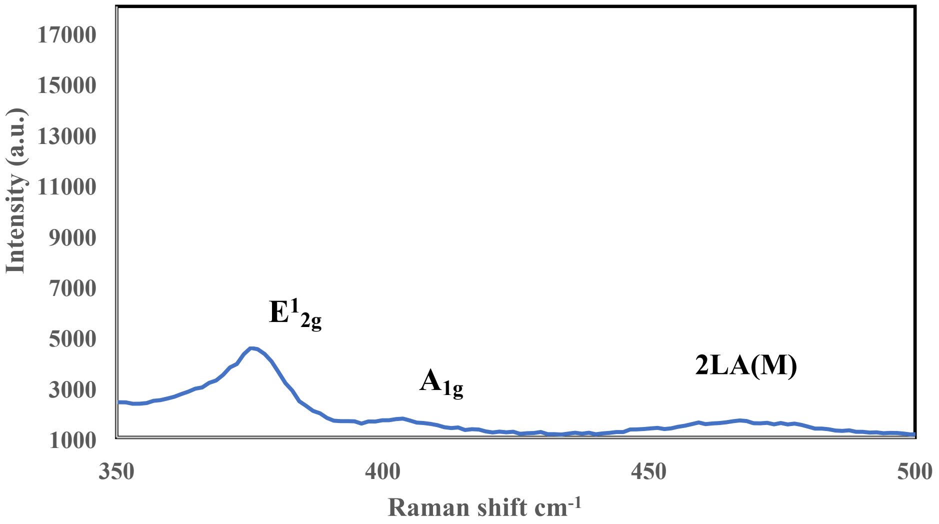

Figure S4: Raman spectra of MoS_2 NFs synthesized by green hydrothermal technique.

S5. Kinetic modelling for the contaminants.

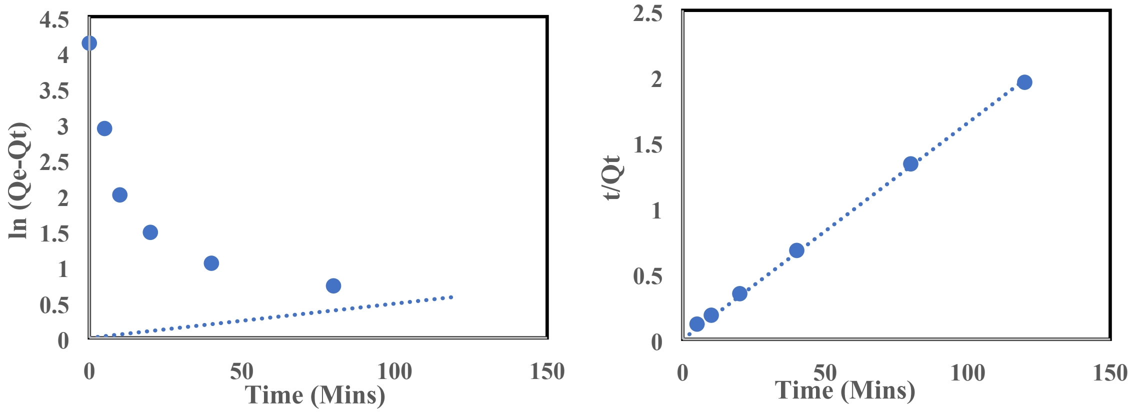

(a) (b)

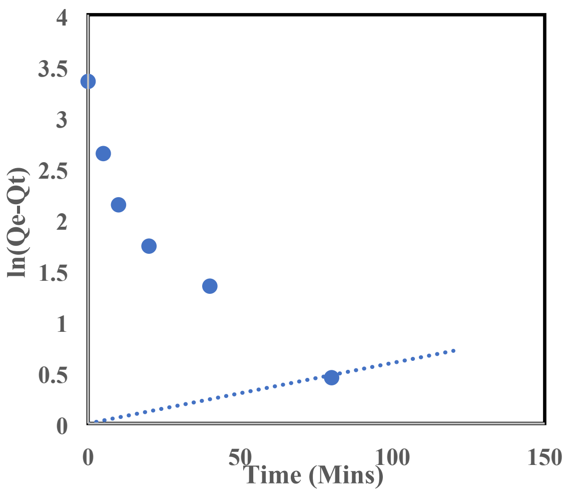

ln(Qe-Qt)
0
0.5
1
1.5
2
2.5
3
3.5
4
0
50
100
150
Time (Mins)

(c)

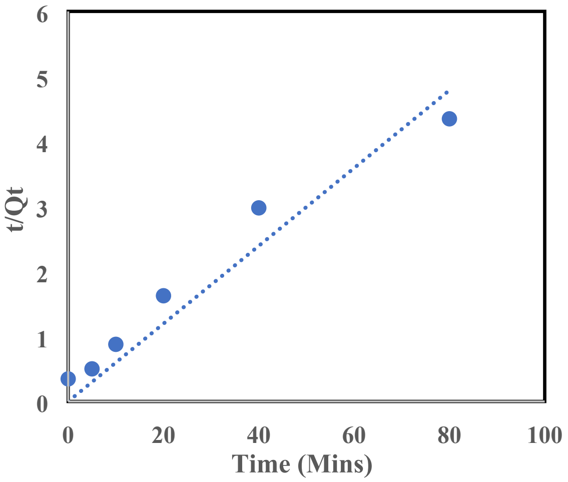

t/Qt
0
1
2
3
4
5
6
0
20
40
60
80
100
Time (Mins)

(d)

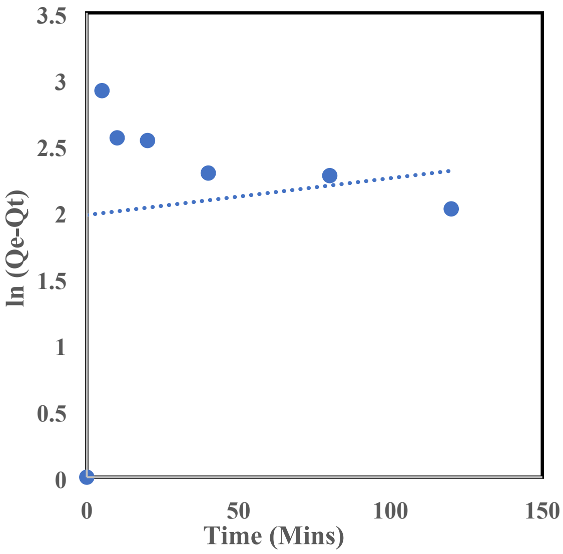

ln (Qe-Qt)
0
0.5
1
1.5
2
2.5
3
3.5
0
50
100
150
Time (Mins)

(e)

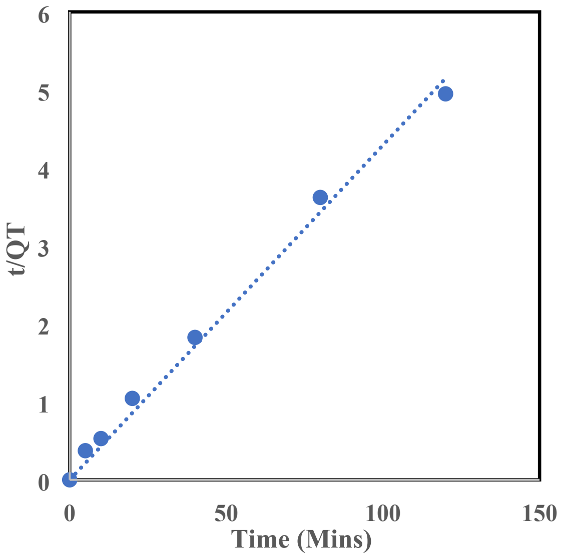

t/QT
0
1
2
3
4
5
6
0
50
100
150
Time (Mins)

(f)

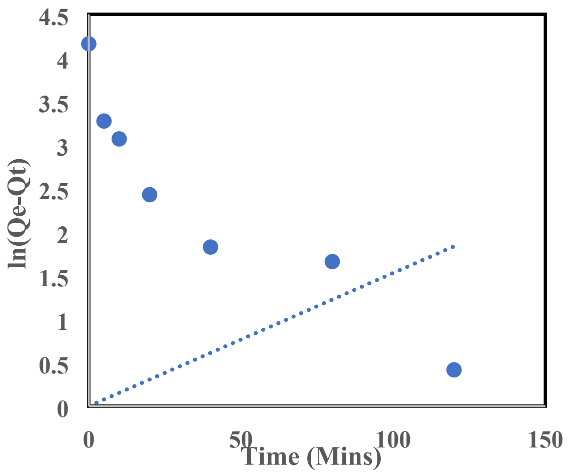

ln(Qe-Qt)
0
0.5
1
1.5
2
2.5
3
3.5
4
4.5
0
50
100
150
Time (Mins)

(g)

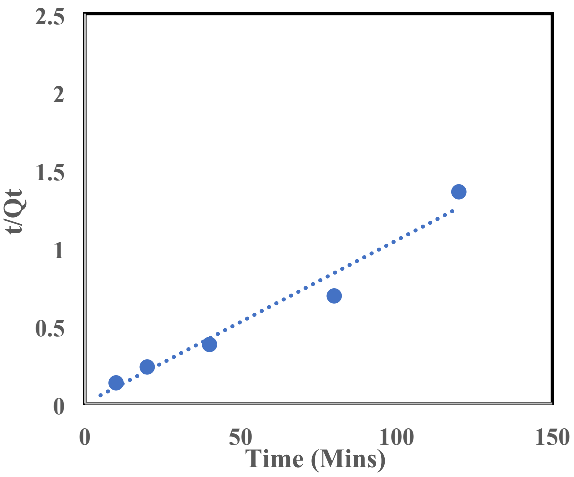

t/Qt
0
0.5
1
1.5
2
2.5
0
50
100
150
Time (Mins)

(h)

Figure S5: a) Psuedo 1st order model for CPN emerging contaminant; b) Psuedo 2nd order model for CPN emerging contaminant; c) Psuedo 1st order model for MeO dye; d) Psuedo 2nd order model for MeO dye; e) Psuedo 1st order model for MeR dye; f) Psuedo 2nd order model for MeR dye; g) Psuedo 1st order model for RD dye; h) Psuedo 2nd order model for RD dye.

S6. Fast kinetics of CPN and RD.

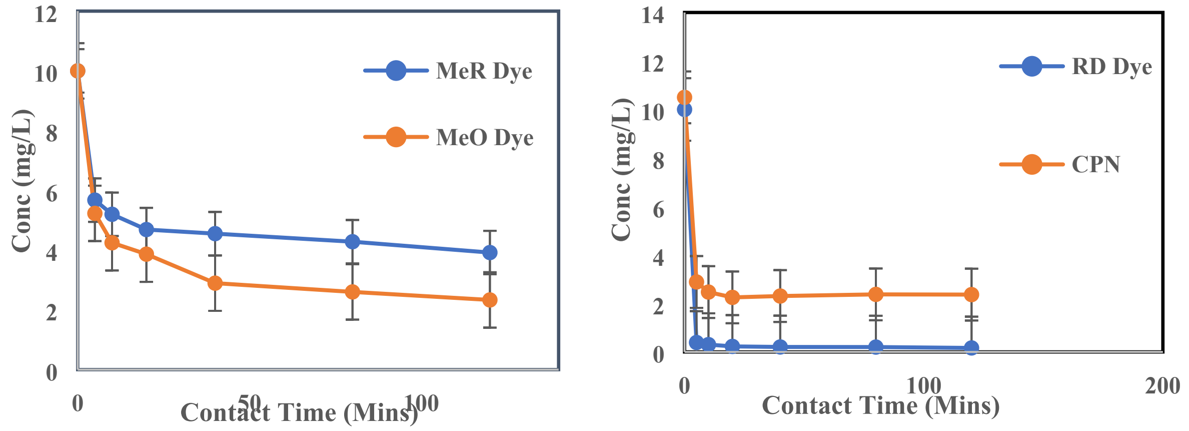

Figure S6: a) Effect of Contact time on adsorption of MeR and MeO (10.0mg/L) onto 1.0 mL of MoS_2 NFs adsorbent solution (5g/L); b) Effect of Contact time on adsorption of CPN and RD (10.0mg/L) onto 0.5 mL of MoS_2 NFs adsorbent solution (5g/L).

S7. SEM result of spent MoS_2 nanoflower

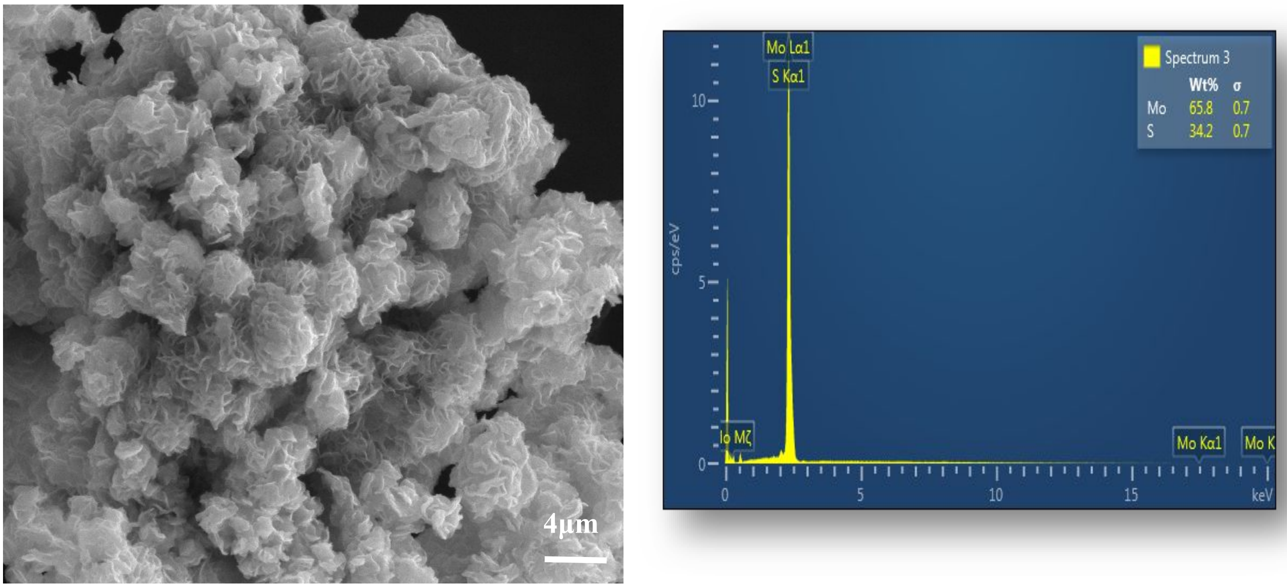

Figure S7: SEM-EDS of spent MoS_2 nanoflower (after 1st cycle): For RD dye.

S8. Thermodynamic modelling of four contaminants

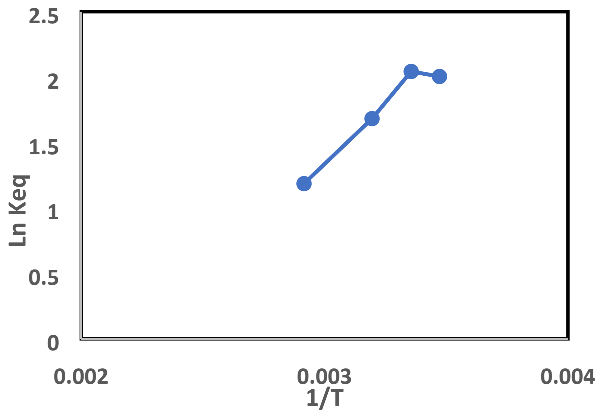

(a)

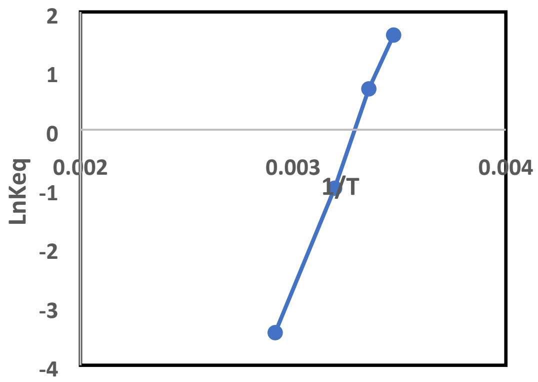

(b)

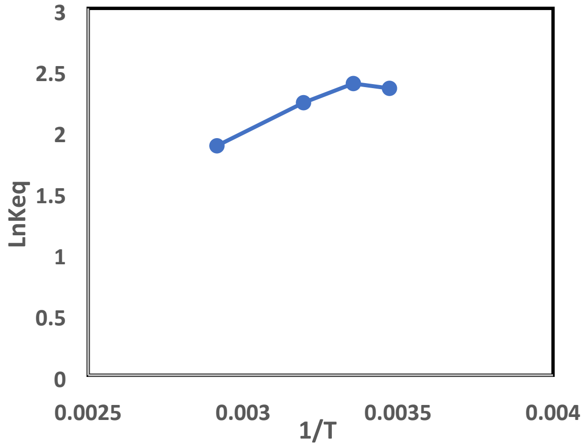

(c)

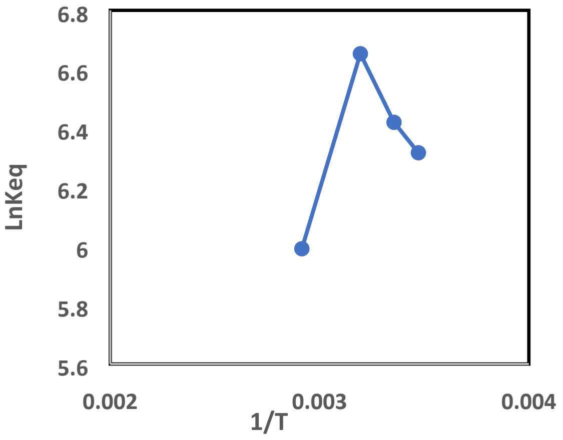

(d)

Figure S8: Thermodynamic modelling with Vant's Hoff concept- a) for MeO dye; b) for MeR dye; c) for CPN contaminant; d) for RD dye.

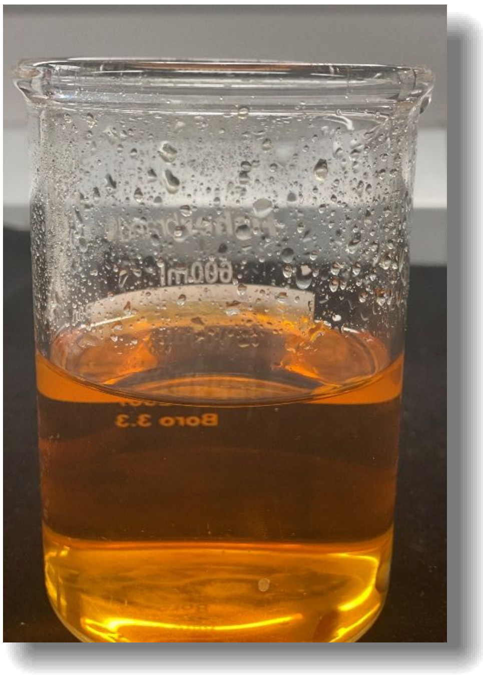

Figure S9: TMDs after washing with DA (at high and low temp).

Dosages (mL)	Removal efficiency of MeO	Removal efficiency of MeR	Removal efficiency of CPN	Removal efficiency of RD
0.5	68	57	78	87
1	73	59	84	93
1.5	78	75.4	80	94
2	**77**	**76**	**85**	**96**

$$R.E.\ (\%) = ((C_{initial} - C_{final}) \times (100)) \div (C_{initial}) \text{---------------} \quad S1$$

Where: $C_{initial}$= initial concentration of the contaminants

$C_{final=}$ final concentration of the contaminants at the equilibrium

Table S1: Summary of Equilibrium study of MoS_2 NFs adsorbents on four different contaminants.

SA[a] (m^2g^{-1})	PV[b] (cm^3g^{-1})	PD[c] (nm)
185.541m^2g^{-1}	2.934 cc/g	39.9 nm

Table S2: Parameters of the MoS_2 NFs sample obtained from the N_2 adsorption-desorption isotherms.

a: BET surface area.

b: Pore volume-DFT desorption cumulative volume of the pores.

c: Pore diameter- average pore diameter determined by the BJH method.

Pore width: 2.769nm

Pore vol: 2.934 cc/g

Total pore volume: 3.142e+00 cc/g for pores smaller than 39.9 nm (Diameter) at P/Po = 0.94945

**Models /Contaminant**	**CPN**	**MeO**	**MeR**	**RD**
**Pseudo 1st order parameters** (Q_e- adsorption Capacity at equilibrium, k_1=pseudo 1st order constant)	R^2= 0.86<br><br>Slope= -0.0236<br>Intercept=2.37<br><br>Q_e= -0.00019681<br>k_1=-0.0001	R^2= 0.958<br><br>Slope= -0.022<br>Intercept= 2.400<br><br>Q_e=11.024<br>k_1=-0.00019	R^2= 0.787<br><br>Slope=0.0005<br>Intercept=2.705<br><br>Q_e= 14.99<br>k_1= -4.99	R^2= 0.86<br><br>Slope= -0.025<br>Intercept= 3.40<br><br>Q_e= 30.26<br>k_1= -0.0001
**Pseudo 2nd order parameters** (Q_e- adsorption Capacity at equilibrium, k_2=pseudo 2nd order constant)	$\underline{R^2=0.999}$<br><br>Slope= 0.0161<br>Intercept=0.0294<br><br>k_2= 130700.4<br>Q_e=62.080	$\underline{R^2= 0.9997}$<br><br>Slope= 0.0348<br>Intercept= 0.181<br><br>k_2=0.0069<br>Q_e= 28.68	$\underline{R^2= 0.996}$<br><br>Slope= 0.040<br>Intercept= 0.196<br><br>k_2= 0.008<br>Q_e= 24.63	$\underline{R^2= 0.9988}$<br><br>Slope=0.0155<br>Intercept= 0.070<br><br>k_2= 0.003<br>Q_e= 64.199

Table S3: Values of fitted kinetic parameters for contaminants adsorption by MoS_2 NFs. R^2 is related to the accuracy of the fitted model. The values of R^2 were underlined to draw attention to the best models.

**CONTAMINANT**	**Freundlich Isotherm Model**	**Langmuir Isotherm Model**	**Temkin Isotherm Model**
**METHYL RED (MeR)**	k_f ({mg/g} {mg/l}$^{1/m}$) = *50.16*<br>m =1/n= *-0.48*<br>*<u>n=-2.08</u>*<br>R^2 = *0.99*	Q_{max} (mg/g) = *18.567*<br>k_L (l/mg) = 0.053<br><br>R^2 = 0.998	k_t (L/mg) = *0.037*<br>B_t (J/mol) = *-13.55*<br><br>R^2 = 0.99
**METHYL ORANGE (MeO)**	k_f ({mg/g} {mg/l}$^{1/m}$) = *44.43*<br>m =1/n= *-0.36*<br>*<u>n=-2.77</u>*<br>R^2 = *0.99*	Q_{max} (mg/g) = *22.49*<br>k_L (l/mg) = *0.044*<br><br>R^2 = *0.98*	k_t (L/mg) = *0.022*<br>B_t (J/mol) = *-11.126*<br><br>R^2 = *0.99*
**CIPROFLOXIN (CPN)**	k_f ({mg/g} {mg/l}$^{1/m}$) = *39.885*<br>m =1/n= *-0.23*<br>*<u>n=-4.34</u>*	Q_{max} (mg/g) = *27.261*<br>k_L (l/mg) = *0.0366*	k_t (L/mg) = *0.007*<br>B_t (J/mol) = *-8.057*

	R^2 = *0.9939*	R^2 = *0.97*	R^2 = *0.99*
**RHODAMINE-B (RD)**	k_f ({mg/g} {mg/l}$^{1/m}$) = *40.47*<br>m =1/n= *-0.011*<br><u>n=-90.90</u><br>R^2 = 0.98	Q_{max} (mg/g) = *41.02*<br>k_L (l/mg) = *-7.2e^{-7}*<br><br>R^2 = 0.92	k_t (L/g) = *2.39e^{-37}*<br>B_t (J/mol) = *-0.47983*<br><br>R^2 = *0.98*

Table S4: Linear adsorption isotherm parameters for all four-contaminant removal by MoS_2 NFs.

**CONTAMINANT**	**Freundlich Isotherm Model**	**Langmuir Isotherm Model**	**Temkin Isotherm Model**
**METHYL RED (MeR)**	k_f= 7.40<br>({mg/g} {mg/l}$^{1/m}$)<br>m =1/n= 1<br>n=-2.083<br>R^2 = 0.99<br>AIC= 34.65	Q_{max} (mg/g) = 28.15<br>k_L (l/mg) = 180616.1<br><br>R_L=5.273E-07<br><br>R^2 = 0.998<br>AIC=26.02	k_t (L/mg) = 0.037<br>B_t (J/mol) = -13.556<br><br>R^2 = 0.99<br>AIC= 62.81
**METHYL ORANGE (MeO)**	k_f=11.04<br>({mg/g} {mg/l}$^{1/m}$)<br><br>m = 1/n= 1<br>n=-4.80<br>R^2 = 0.99<br>AIC=31.42	Q_{max} (mg/g) = 31.135<br><br>k_L (l/mg) = 31460.04<br><br>R_L= 3.027E-06<br><br>R^2 = 0.98<br>AIC=20.719	k_t (L/mg) = 0.022<br>B_t (J/mol) = -11.126<br><br>R^2 = 0.99<br>AIC=66.93
**CIPROFLOXIN (CPN)**	k_f= 16.744<br>({mg/g} {mg/l}$^{1/m}$)<br>m =1/n= 1<br>n=-4.34<br>R^2 = 0.9939<br>AIC= 32.865	Q_{max} (mg/g) = 27.26<br>k_L (l/mg) = 0.0366<br><br>R_L= 0.72<br>R^2 = 0.97<br>AIC=16	k_t (L/mg) = 0.724<br>B_t (J/mol) = -2369096.7<br><br>R^2 = 0.99<br>AIC= 42.74
**RHODAMINE-B (RD)**	k_f= 318.08<br>({mg/g} {mg/l}$^{1/m}$)<br>m =1/n= 1<br>n=-90.90<br>R^2 = 0.98<br>AIC= 38.523	Q_{max} (mg/g) = 41.56<br>k_L (l/mg)=12773021.63<br><br>R_L= 7.45619E-09<br><br>R^2 = 0.92<br>AIC=2.72	k_t (L/g) = 12.71<br><br>B_t (J/mol) = -3882.8812<br><br>R^2 = 0.98<br>AIC=41.84

Table S5: Non-Linear adsorption isotherm parameters for all four-contaminant removal by MoS_2 NFs.

Adsorbent/ Contaminant	Temperature (°C)	Temperature (K)	Keq (-)	ΔG° (KJmol-1)	ΔH°(KJ mol-1)	ΔS°(Jk-1mol-1)	R^2
TMD/MeO DYE	15	288	7.428571	-4.80163487	-13.2725	-28.5504	0.943416
	25	298	7.71769	-5.0629557			
	40	313	5.38233	-4.37995641			
	70	343	3.276003	-3.38389831			

Table S6: Thermodynamics parameter for removal of MeO dye by MoS_2 NFs.

Adsorbent/ Contaminant	Temperature (°C)	Temperature (K)	Keq (-)	ΔG° (KJmol-1)	ΔH°(KJmol-1)	ΔS°(Jk-1mol-1)	R^2
TMD/MeR DYE	15	288	4.990786372	-3.8492732	-76.4522	-251.73	0.998352
	25	298	2.00128599	-1.7189146			
	40	313	0.370220072	2.585777447			
	70	343	0.03187098	9.827134475			

Table S7: Thermodynamics parameter for removal of MeR dye by MoS_2 NFs.

Adsorbent/ Contaminant	Temperature (°C)	Temperature (K)	Keq (-)	ΔG° (KJmol-1)	ΔH°(KJ mol-1)	ΔS°(Jk-1mol-1)	R^2
**TMD/CPN**	15	288	10.44440623	-5.61749681	-7.5889	-6.18011	0.903308
	25	298	10.86304763	-5.90991825			
	40	313	9.289036545	-5.80005678			
	70	343	6.528954625	-5.35049687			

Table S8: Thermodynamics parameter for removal of CPN by MoS_2 NFs.

Adsorbent/ Contaminant	Temperature (°C)	Temperature (K)	Keq (-)	ΔG° (KJmol-1)	ΔH°(KJ mol-1)	ΔS°(Jk-1mol-1)	R^2
**TMD/RD DYE**	15	288	553.7689243	-15.1250223	-4.91046	36.87038	0.268957
	25	298	614.0105945	-15.9060424			
	40	313	774.9317507	-17.3123965			
	70	343	399.8461538	-17.0847744			

Table S9: Thermodynamics parameter for removal of RD dye by MoS_2 NFs.
